# Direct Anterior Approach in Total Hip Arthroplasty for Severe Crowe IV Dysplasia: Retrospective Clinical and Radiological Study

**DOI:** 10.3390/medicina60010114

**Published:** 2024-01-07

**Authors:** Cesare Faldini, Leonardo Tassinari, Davide Pederiva, Valentino Rossomando, Matteo Brunello, Federico Pilla, Giuseppe Geraci, Francesco Traina, Alberto Di Martino

**Affiliations:** 1 I Orthopedic and Traumatology Department, IRCCS Istituto Ortopedico Rizzoli, 40136 Bologna, Italy; cesare.faldini@ior.it (C.F.); leonardo.tassinari@ior.it (L.T.); davide.pederiva@ior.it (D.P.); valentino.rossomando@ior.it (V.R.); matteo.brunello@ior.it (M.B.); federico.pilla@ior.it (F.P.); giuseppe.geraci@ior.it (G.G.); 2 Department of Biomedical and Neuromotor Science-DIBINEM, University of Bologna, 40126 Bologna, Italy; francesco.traina@ior.it; 3 Orthopedics-Traumatology and Prosthetic Surgery and Hip and Knee Revision, IRCCS Istituto Ortopedico Rizzoli, 40136 Bologna, Italy

**Keywords:** direct anterior approach, hip dysplasia, Crowe IV, total hip arthroplasty, femoral osteotomy, complications

## Abstract

*Background and Objectives*: total hip arthroplasty (THA) for Crowe IV hip dysplasia poses challenges due to severe leg shortening, muscle retraction and bone stock issues, leading to an increased neurological complication, and revision rate. The direct anterior approach (DAA) is used for minimally invasive THA but its role in Crowe IV dysplasia is unclear. This retrospective study examines if DAA effectively restores hip biomechanics in Crowe IV dysplasia patients with <4 cm leg length discrepancy, managing soft tissue and yielding functional improvement, limb length correction, and limited complications. *Materials and Methods*: 19 patients with unilateral Crowe IV hip osteoarthritis and <4 cm leg length discrepancy undergoing DAA THA were reviewed. Surgery involved gradual soft tissue release, precise acetabular cup positioning, and stem placement without femoral osteotomy. *Results*: results were evaluated clinically and radiographically, with complications recorded. Follow-up revealed significant Harris Hip Score and limb length discrepancy improvements. Abductor muscle insufficiency was present in 21%. The acetabular component was accurately placed, centralizing the prosthetic joint’s rotation. Complications occurred in 16% of cases, including fractures, nerve issues, and infection. DAA in THA showcased positive outcomes for hip function, limb length, and biomechanics in Crowe IV dysplasia. *Conclusions*: the technique enabled accurate cup positioning and rotation center adjustment. Complications were managed well without implant revisions. DAA is a viable option for Crowe IV dysplasia, restoring hip function, biomechanics, and reducing limb length discrepancy. Larger, longer studies are needed for validation.

## 1. Introduction

**Figure 1:**
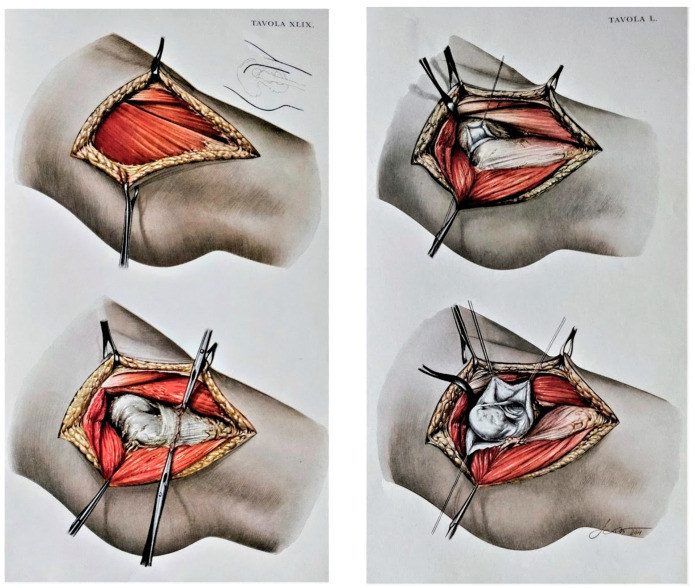
In the original anatomic drawings by Remo Scoto, made on commission by Vittorio Putti himself, the steps of reduction of the hip dislocation through DAA are outlined; the drawings show how DAA is powerful in managing all the pathological elements of DDH in terms of muscular and bony components; moreover, it is possible to fully expose and manage capsule, ligamentum teres at the true acetabulum, and to isolate and section the retracted soft tissues including the psoas muscle (reproduced from the book “Anatomy of congenital dislocation of the hip” by V. Putti, G. Faldini and E. Pasquali, Bologna, Licinio Cappelli Editore, 1935, by permission of Istituto Ortopedico Rizzoli [12]).

Total hip arthroplasty (THA) surgery is a highly effective option for restoring the inflamed state of the hip joint, especially in cases of Crowe IV hip dysplasia. However, achieving hip biomechanics restoration and optimal component positioning in THA for Crowe IV hip dysplasia poses challenges due to severe muscle retraction and poor bone stock resulting from pathologic anatomy [1,2]. Various techniques have been proposed to address these challenges, including proximalization of the center of rotation (COR) for a high hip center THA, which may sacrifice physiological hip biomechanics [3], femoral shortening osteotomy, and extensive soft tissue release to reduce the hip at the true acetabulum [4]. Despite the complexity, many authors argue that positioning the cup at the true acetabulum is associated with better long-term survival rates [5,6,7,8,9], even though it requires a more intricate surgical exposure to facilitate implant reduction at the end of the surgery. Historically, Heuter, Smith-Petersen, and Putti described approaches to the dysplastic hip joint through the sartorius and tensor fasciae latae intermuscular space. Accessing the joint from the anterior allows the surgeon to easily manage redundant capsular tissue and address all pathological alterations of soft tissues by performing a release of the tensor fasciae latae, medius gluteus, rectus anterior muscle, and iliopsoas muscle (Figure 1) [10,11,12]. At present, a modified version of the original direct anterior approach (DAA) to the hip joint is utilized for minimally invasive THA [12,13,14,15,16]. This approach can be extended proximally and distally to enhance exposure to the pelvis and femur, if necessary. The DAA has gained popularity in THA due to its ability to address hip muscles through an internervous and intermuscular approach. Benefits of this approach include faster healing, reduced pain, and a lower postoperative dislocation rate compared to traditional methods [17,18]. Consequently, the DAA appears to be the most effective approach for THA, at least theoretically.

Despite the theoretical advantages associated with the use of DAA in Crowe IV dislocated hips, its use has not gained popularity for complex surgery. Only a restricted amount of literature has discussed the role of DAA for THA in DDH patients [19,20,21,22], but the heterogeneity in patients’ selection and the tendency to perform an adjunctive femoral shortening osteotomy compromise the ability to define the power of DAA alone in such patient populations. The novelty of this study is to report the results of an anterior technique THA in patients suffering from Crowe IV with moderate LLD, acting only on the soft tissues, in a tailored way for the single patient, avoiding the femoral shortening ostetomy. The rationale of the present study is to evaluate the effectiveness of DAA in a homogeneous population of patients with unilateral Crowe IV hip osteoarthritis (OA) requiring a THA. The aims of this study are to investigate (1) whether THA through DAA, with accurate cup placement in the true acetabulum, is a viable and functional approach for maintaining abductor muscles, enhancing hip function, and addressing limb length discrepancy (LLD) without femoral osteotomy. (2) The extent to which this procedure consistently reestablishes the physiological center of rotation of the hip joint at the true acetabulum. (3) The intra and post-operative complications associated with this technique, along with the strategies employed for their management.

## 2. Materials and Methods

### 2.1. Patients

This study was approved by the institutional review board and ethical committee, and it was entirely conducted at the authors’ Institution (code 347/2021/Oss/IOR). We retrospectively reviewed 19 patients with unilateral hip OA secondary to Crowe IV DDH who underwent a THA through a DDA between January 2016 and December 2020. The dysplastic hips were classified according to the original Crowe classification [23]. Exclusion criteria included patients with neurological diseases compromising ambulation, flaccid (e.g., poliomyelitis) or spastic (e.g., infantile cerebral palsy) paralysis, patients operated on for femoral neck fractures, patients with a radiological LLD greater than 4 cm, or bilateral DDH. The rationale behind the exclusion of patients with LLD above 4 cm is due to our treatment algorithm; indeed, in the major LLD, we perform femoral shortening osteotomy or a progressive distraction and THA at a later stage. All the patients underwent unilateral cementless THA using a DAA with progressive soft tissue release [24], cup positioning at the true acetabulum, and straight or conical stem placement without femoral shortening osteotomy. Surgery was performed by a single experienced surgeon in DAA in a single high-volume center. The implants used for the acetabulum were in all patients a hemispheric cup, coated with 3D-printed porous titanium, and at least two fixation screws were also implanted. Meanwhile, in 8 (42.1%) of the 19 operated patients a conical, short, fixed stem, coated with hydroxyapatite, was used because of the elevated anteversion of the proximal femur, and in the remaining 11 patients (57.9%) a straight, fixed, triple tapered stem, coated in hydroxyapatite, was implanted. In all patients coupling was ceramic-on-ceramic, using a large femoral head diameter. Percutaneous tenotomy at the tendon of the adductor was performed in 4/19 (25%) patients in which a limitation of the hip abduction was observed.

### 2.2. Surgical Technique

**Figure 2:**
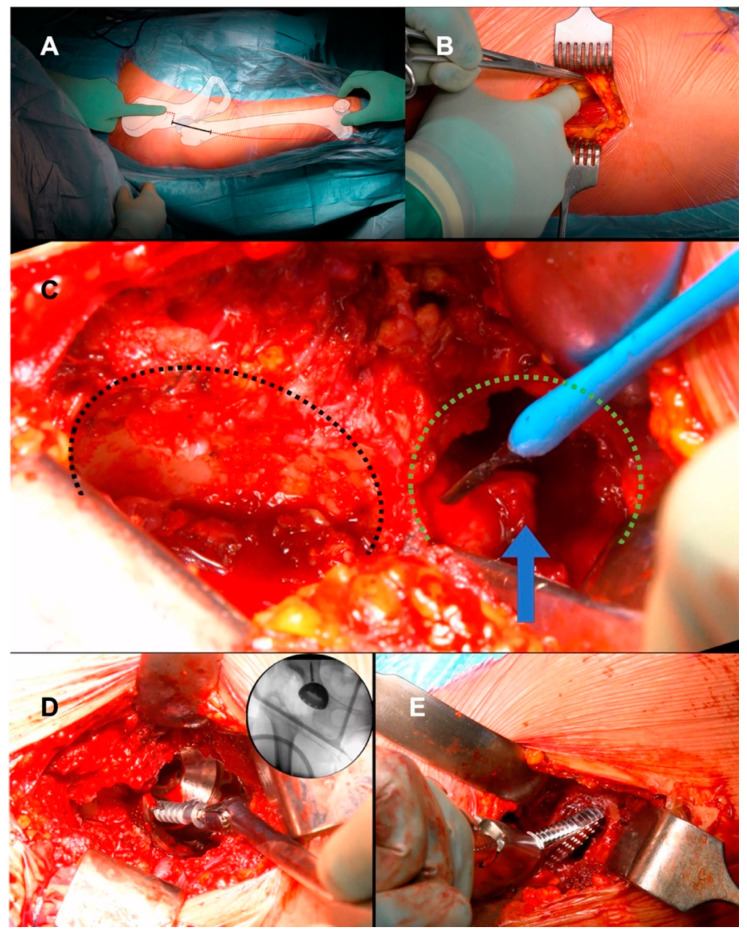
(**A**) Intraoperative photograph of the right hip of a 57-year-old woman with an illustration overlay showing anatomic landmarks for the anterior approach to the hip. The anterior superior iliac spine, the tip of the greater trochanter, the fibular head, and the patella should be included in the surgical field to estimate femoral shaft orientation via palpation of the femoral condyles, which guides neck resection and femoral component positioning. The skin incision begins 2 cm distal and lateral to the anterior superior iliac spine and is directed toward the fibular head for approximately 7–8 cm. (**B**) Elevation of the medial side of the aponeurosis with the use of Kocher forceps and separation of the belly of the tensor fascia latae muscle. A finger is used to develop the intermuscular space between the tensor fascia latae and the sartorius muscles, with the sartorius and the rectus femoris displaced medially and the tensor fascia latae displaced laterally. (**C**) After capsulotomy, the relationship between the false acetabulum (black dashed line) and the true acetabulum (green dashed line) is outlined. The false acetabulum is flat and wide and lies on the surface of the iliac bone. The true acetabulum is small and deficient and is covered by hypertrophic capsular tissue and pulvinar (arrow). Using electrocautery, soft tissue is carefully cleared to achieve complete exposure of the true acetabulum. (**D**) After impaction of the cup in the true acetabulum, two cancellous screws are positioned in the safe zone of the ilium to promote fixation. (**E**) At the femoral level, broach insertion should be performed in line with the femoral canal (15° of anteversion). (Figure drawn by Leonardo Tassinari, MD for this manuscript).

**Figure 3:**
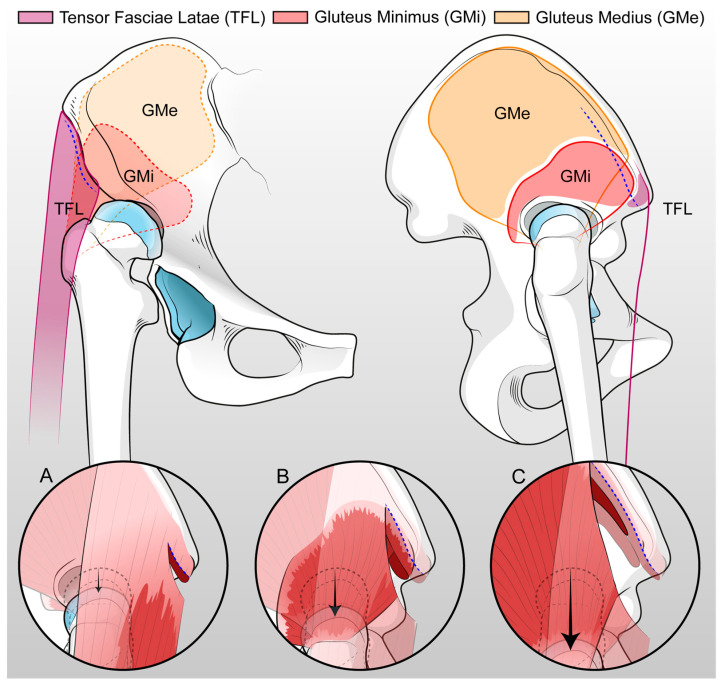
Anterior and lateral view of the hemi-pelvis with the representation of the progressive muscular release in order to obtain the reduction of the femur in the true acetabulum, avoiding femoral shortening osteotomy. The first step includes the detachment of TFL about 1 cm from SIAS (**A**) and the second step consists of the partial release of both TFL and GMi deeply into the iliac crest about 2 cm from SIAS (**B**). Lastly, the third step includes the partial release of the GMe (**C**) (figure drawn by Leonardo Tassinari, MD for this manuscript).

Surgery was performed by DAA in all patients as shown in Video S1. Briefly, the patient lay on a dedicated traction table. The incision started 2 cm distal and 2 cm lateral to the superior inferior iliac spine, and it was extended distally about 7 cm crossing the inguinal fold (Figure 2). The intermuscular plane between the sartorius muscle and the rectus femoris medially, and the tensor fascia latae (TFL) laterally, was identified and developed. After capsulotomy, the femoral neck was exposed: in dysplastic hips, the local anatomical landmarks are not very reliable, therefore, external landmarks were used to correctly perform the neck osteotomy, including femoral shaft positioning and orientation of the patella. After neck osteotomy, a full release of the joint capsule at both the femoral and acetabular sides was required to reduce the hip at the true acetabulum. At the same time, a partial release of the origin of TFL, gluteus minimus (GMi), and gluteus medius (GMe) muscles from the iliac wing, and a release of the iliopsoas tendon at the insertion onto the lesser trochanter, were tailored based on the pathological anatomy of the patients. Release of the muscles was performed starting from the ASIS and extended gradually proximally and deeply to the iliac crest to obtain proper lengthening without compromising the entire origin of the muscles or detaching their insertion at the greater trochanter (Figure 3). The removal of a thick and redundant capsular tissue exposes the false and true acetabulum. The latter is classically hypoplastic and shallow and hosts a hypertrophic pulvinar. Progressive reaming of the true acetabulum was started by using a small reamer to medialize the center of rotation and develop the bone cavity; subsequently, ream size was increased until good stability and sufficient size to host a cup were achieved. Fluoroscopy was then performed to check the correct positioning of the cup in the true acetabulum, before the screw’s insertion. In most patients, press fit cup benefited from adjunct screw fixation in the posterior–superior quadrant to increase primary stability, as the bone in the true acetabular area is typically osteopenic due to disuse. To expose the femoral canal through DAA, the limb must be externally rotated, extended, and adducted. The direction of the femoral canal was identified through the sequential use of a chisel and a curved spoon, and then broaching was performed. After the final components were in place, the reduction of the hip was achieved by traction of the lower limb in a close sequence of abduction, internal rotation, and flexion. During the closing, when possible, the TFL, GMi, and GMe were reattached, using stiches, depending on the lengthening obtained. The anterior capsule, usually hypertrophic and redundant, was removed during the true acetabulum exposure or the closure. Percutaneous tenotomy of hip adductors could be performed before surgery or after wound closure if adduction contracture due to limb lengthening limited joint movement as shown in Video S2.

### 2.3. Post-Operative Management 

Postoperative rehabilitation protocol depended on the presence of residual adductor contracture and on the entity of limb lengthening. Physical therapy was aimed at reducing contractures in flexion and adduction, which usually regressed 3 to 6 months after surgery. Ambulation with partial weight bearing (25%) on the operated limb was allowed from the day after surgery. One month postoperatively, 50% weight bearing was granted, while the full load was allowed 8 weeks after surgery. Patients were followed up clinically and radiographically at 1 month, 3 months, 1 year, and then yearly, for a minimum of 2 years postoperative follow-up. 

### 2.4. Clinical Evaluation

Clinical scores and study parameters included the Harris Hip Score (HHS), the evaluation of apparent LLD, and the presence of a Trendelenburg sign and gait. The Harris Hip Score (HHS) [25] in the Italian-validated version was used to quantify parameters such as pain, function, and range of motion of the operated hip on a numerical scale; according to this score, results below 70 are considered poor, between 70 and 79 are discrete, between 80 and 89 are good, and between 90 and 100 are excellent. All patients were tested for LLD before and after surgery, A negative LLD with respect to the contralateral side was expressed with the sign “−”, and a positive LLD with the sign “+”. Apparent LLD was evaluated by block test using lifts with a 2 mm thickness progression, until the patient felt leg length equality (LLE) [26,27]. All the patients performed a postoperative antero-posterior radiographic control to check implant position.

### 2.5. Radiographic Evaluation

Radiographic analysis was performed by three independent hip surgeons to keep systematic error rate low. Pelvic tilt and rotation have been verified before further assessments, the tilt was checked measuring the distance between the midportion of the sacrococcygeal joint and the upper border of the symphysis pubis, and the rotation was considered neutral if the coccyx was in line with the symphysis pubis. Parameters included the evaluation of the COR of the native femoral head and THA, true LLD before and after surgery, and implant osteointegration according to Moore for the acetabular component [28] and according to Engh [29] for the femoral component. COR was measured on the horizontal and vertical axis, taking as a reference the apex of the inter-tear drop line on the anteroposterior radiograph of the pelvis [30]; true LLD was measured as the distance between proximal crossing of the femur and lesser trochanter and the bottom part of the pelvic teardrop on an anteroposterior radiograph [31]. 

### 2.6. Statistical Analysis 

Distribution of variables was reported using means and ranges for normally distributed data. Data were tested for normality using the Shapiro–Wilk test. The paired *t*-test was used to compare pre- and post-operative findings. *p*-values < 0.05 were considered significant. SPSS 17.0 statistical analysis software (SPSS Inc., Chicago, IL, USA) was used to perform statistical analysis.

## 3. Results

### 3.1. Demographics

The patient population included 15 females and 4 males, with an average age at surgery of 55 years (range 32–71); the average BMI was 26.4 (range 19–34). The average follow-up was 33.4 months (range 24–49).

### 3.2. Clinical Results

At the last available follow-up, an average HHS improvement of 44.8 points was found, with preoperative values averaging 44.6 points (38–56), and a postoperative average of 89.4 points (82–96) (*p* < 0.001) (Table 1). Abductor muscle insufficiency with positive Trendelenburg gait and sign, which preoperatively was present in all the patients, was observed in 4 out of 19 patients (21%) at the last clinical evaluation. All patients presented an LLD before surgery. The sensation of LLE was obtained at the first follow-up (4 weeks) only in 5 patients (26.3%), in 6 patients (31.5%) at 3 months, in 10 patients (52.6) % at 1 year, and in 12 (63.1%) at the last available evaluation (*p* < 0.001). Apparent LLD at the operated limb decreased from a preoperative average, −3.5 cm (range 2.5–4.3 cm), to −1.2 cm (range 0.5–2.4 cm) at 4 weeks after surgery (*p* < 0.001); at 1 year, it averaged −0.7 cm (0.4–1.4 cm) and decreased up to −0.4 cm (range 0.2–0.8 cm) at the last follow-up. 

### 3.3. Radiographic Results

**Figure 4:**
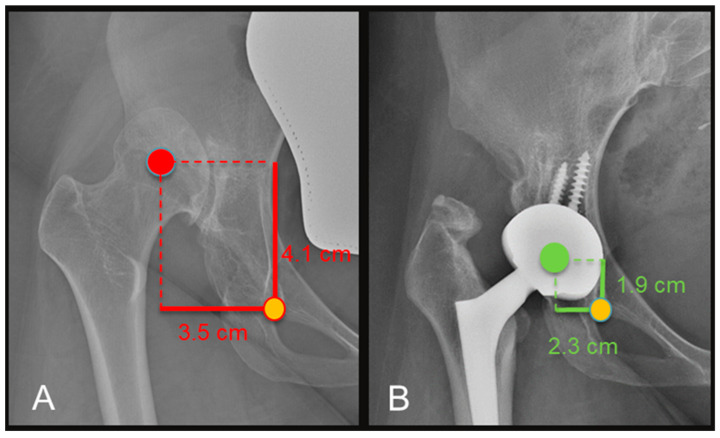
Pre-operative (**A**) and 18 months (**B**) X-rays showing medialization and distalization of the COR with a decrease in both horizontal and vertical distance from the teardrop.

**Figure 5:**
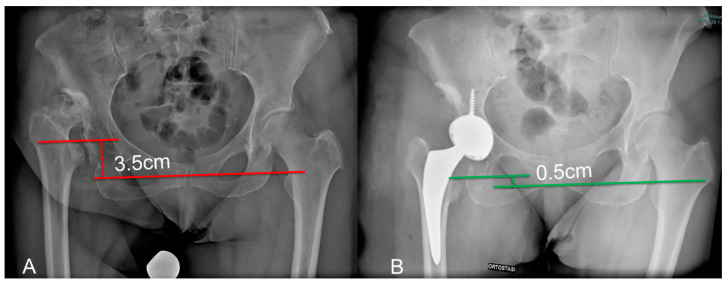
Pre-operative (**A**) and post-operative (**B**) X-rays showing a reduction in anatomical LLD referenced to the lesser trochanter.

The acetabular component was positioned at the true acetabulum in all the patients, with distalization and medialization of the COR of the prosthetic joint. On the horizontal plane, the distance between the COR and the inter-tear drop line significantly decreased from a preoperative average of 4.4 cm (range 4–4.8) to a postoperative value of 2.4 cm (range 1.3–3.4) (*p* < 0.0003) (Figure 4). The distance between the COR and the apex of the radiographic drop was significantly reduced in the vertical plane (*p* < 0.002), passing from preoperative values of 4.6 cm (range 2.8–6.6) to average postoperative values of 1.9 cm (range 1.1–2.4). True LLD significantly reduced (*p* < 0.0001) from a preoperative average of −3.4 cm (range 1.6–4 cm) at the operated limb to −0.8 cm (range 0.3–1.1 cm) after surgery (Figure 5). At the last available follow-up, no significant loosening of the components or osteolysis was observed. The radiographic evaluation of the acetabular component according to Moore showed at least three signs of osteointegration in all the postoperative radiographs. The radiographic study of the stem according to Engh showed an average total value of 22 out of 27 (range 18.5–27), with an average fixation score equal to 7.5 (range 5–10) and a stability score of 14.5 (range 13.5–17), strongly predictive of osteointegration of the prosthetic components.

### 3.4. Complications

Our case series was complicated by one intraoperative subtrochanteric fracture, one transient paralysis of the femoral nerve, and one superficial surgical wound infection, accounting for a complication rate of 16%. The single superficial wound infection was from the main incision and was treated with a superficial wound debridement and oral antibiotics for 15 days with complete resolution. The subtrochanteric fracture was managed intraoperatively by metal wiring, which required a distal extension of the surgical incision of about 4 cm. After wiring, use of crutches and a delayed load on the operated limb was required; full load was granted 3 months after the surgery. The patient with transient paralysis of the femoral nerve was a female patient and was treated through a dedicated rehabilitation program with electrostimulation of the quadriceps muscle and targeted strengthening and stretching exercises. The deficit was fully recovered by the sixth postoperative month. Two out of nineteen (10.5%) patients underwent percutaneous adductor tenotomy, respectively, at 6 and 8 months after surgery, because of a severe adductor muscle contracture not improved by postoperative physical therapy; clinically, patients complained of a deficit of hip abduction and symptomatic apparent LLD. None of those patients received intraoperative tenotomy of the adductors. The procedure ensured a rapid improvement of the limitation in hip abduction, and a sensation of LLE. Two patients showed postoperative true LLD values > 1 cm, with limp during ambulation. Both benefited from the use of a shoe lift at the operated limb, achieving a sensation of LLE. No patient required implant revision at the latest available follow-up.

## 4. Discussion

In the current study, patients undergoing THA through the DAA for the treatment of unilateral osteoarthritis secondary to Crowe IV dislocated hip with up to 4 cm limb shortening reported favorable clinical and radiographic outcomes, along with an acceptable complication rate. The management of pathological anatomy through DAA facilitated effective and progressive release of soft tissue contracture, characteristic of the neglected dislocated hip. Tailored soft tissue release for individual patients allowed the restoration of the physiological COR and hip biomechanics, reducing the need for ancillary procedures such as femoral shortening osteotomy. The prosthetic hip’s reduction at the true acetabulum was supported by the complete excision of the hypertrophic capsule. Through DAA, the Gluteus Medius (GMi) and Gluteus Maximus (GMe) muscles, instead of being detached from the greater trochanter, could be released from the origin at the iliac wing. Similarly, the Iliopsoas and anterior rectus muscles, dysfunctional and retracted in patients with a chronically dislocated hip, were easily exposed and released when necessary, supporting the proximal femur’s reduction toward the true acetabulum [32]. Preserving the insertion of abductor muscles at the greater trochanter promoted positive clinical outcomes with a significant improvement in gluteal function, lateral, and vertical offset restoration [33].

Originally developed by Heuter for managing infections and traumas at the hip [34], the DAA was subsequently used for the open reduction of dislocated dysplastic hips [35]. This approach enables the management of all pathological elements of DDH by performing a tailored soft tissue release based on the severity of the anatomical picture [36]. A notable feature of DAA is its versatility, allowing extension both proximally to expose the false acetabulum and distally to perform a femoral osteotomy or wiring of an intraoperative femoral fracture [15]. In our high-volume department, almost all primary and secondary coxarthrosis cases are treated using DAA. Conditions such as obesity and muscular males are still addressed using this technique. In obese patients, the “bikini” incision is preferred for better postoperative wound management [37]. The implementation of DAA in THA surgery for Crowe IV patients is relatively recent, and reports are limited to case series due to the rarity of the disease and the limited adoption of DAA among hip surgeons. Previous studies, such as that by Oinuma et al. [19], reported satisfactory outcomes in Crowe IV DDH patients undergoing THA replacement for osteoarthritis, with a femoral shortening osteotomy performed in each patient. Another study by Viamont-Guerra et al. [21] reported satisfactory medium- to long-term results in DDH patients undergoing THA through DAA, with four intraoperative femur fractures (21%) and a revision rate of 10% at an average follow-up of 8 years. In our experience, at an average follow-up of 2.7 years, no revisions were necessary, good implant osteointegration was observed, and no patients required femoral shortening osteotomy or femoral head autologous bone grafting to manage the acetabular bone defect. The clinical outcomes were favorable in terms of the Harris Hip Score (HHS), and the intraoperative femoral fracture rate was 5.3%.

Comparing the DAA approach to traditional THA approaches, including lateral, posterolateral, and posterior approaches, has shown excellent outcomes in DDH patients, both in terms of complications and long-term revision rates [38,39,40,41,42]. Traditional techniques are associated with some degree of muscle damage, with postoperative dislocation rates of up to 16.6% [2]. Type IV DDH is linked with hip muscular weakness, potentially predisposing individuals to surgical dislocation [9].

The main limitations of the current study include its retrospective nature, a low sample size, and the absence of a control group with a non-dysplastic patient cohort. Moreover, a larger number of patients and a longer follow-up would be required to confirm the current findings regarding implant failure and long-term complications, as well as for comparison with results in a non-dysplastic population. However, the exclusion of patients with bilateral DDH and LLD above 4 cm makes our conclusions highly specific for a subset of DDH patients. Patients in our study population exhibited positive results in terms of the recovery of apparent LLD, with the resolution of abduction insufficiency and limp due to gluteal insufficiency in most cases. Apparent LLD exhibited a slow but progressive improvement in patients with adduction contracture; rehabilitation therapy, consisting of stretching the adductor muscles, allowed for the achievement of fully active and passive abduction. Two patients with persistent adduction contracture at the operated limb underwent subsequent percutaneous adductor tenotomies at 6 and 8 months after the primary surgery. These results underscore the importance of the correct management of soft tissues to achieve both the restoration of the COR at the true acetabulum and functional results during rehabilitation. The contracture of the adductor muscles is usually managed percutaneously in pediatric patients with a dislocated hip and in the adult population affected by Crowe IV DDH. Given the limitation of joint range of movement due to arthritic degeneration at the false acetabulum, adductor contracture may be underestimated and consequently not managed during THA surgery [9]. In patients with a dysplastic or dislocated hip, adductor contracture significantly alters hip biomechanics, maintaining a condition of forced adduction; after THA, if persistent, adduction contracture increases the shear stress at the implant–bone interface [43], determining limited abduction, increased apparent LLD, the risk of loosening of the acetabular component, and the risk of implant dislocation. Percutaneous adductor tenotomy is sufficient in most patients to manage the residual adduction at the end of THA surgery; alternatively, it is performed preoperatively in patients with an elevated suspicion of adductor contracture [8]. In our experience, we do not perform this procedure routinely on all dysplastic patients. In the case of modest contractures, prosthetic replacement alone is often sufficient to allow acceptable degrees of abduction, especially when there is significant impingement between the femur and ilium. In the case of severe contractions or post-operative intolerance by the patient, this procedure is then performed.

Surgery was aimed at positioning the COR at the true acetabulum in all 19 patients. As reported by Komiyama et al. in a retrospective study of 1079 prosthetic implants in dysplastic patients [30], COR positioning is the main determinant of implants’ mechanical complications. Placement of the acetabular cup at the true acetabulum in Crowe IV DDH patients showed better survival rates when compared to COR at the false acetabulum [5]; in fact, positioning the acetabular component at the false acetabulum is associated with an increased risk of aseptic loosening [6,7]. In a study of 49 patients with a 30-year follow-up, Watts et al. [44] showed 68% of failures of the acetabular component in case of proximalization of the COR, compared to 35% of failures in the case of COR at the true acetabulum. Similarly, Linde and Jensen [45] reported 42% of cases of aseptic loosening of the acetabular component when it was implanted at the false acetabulum in a series of 123 THAs in DDH. Stans et al. [46], at an average 16.6-year follow-up (range 5–23), reported a failure rate of the acetabular component in 83.3% of patients when the COR was placed outside the true acetabulum, mostly because of acetabular loosening. However, other authors suggest that a high hip COR in DDH patients showed no significant differences in terms of implant survival when compared to cups placed at the true acetabulum [5,47,48].

Montalti et al. [3] reported the results of a series of 80 modular neck Total Hip Arthroplasties (THAs), 37 of which were performed on Crowe IV hips. At an average 15-year follow-up, they documented excellent clinical and radiographic outcomes, with revision required in only two patients—one due to aseptic cup loosening and the other due to aseptic loosening of the femoral component. Achieving the correct leg length by placing the cup at the false acetabulum without the use of long modular necks is seldom possible. Moreover, if a high hip center THA is performed, a ceramic-on-ceramic coupling is required, limiting the use of polyethylene lips in case of suboptimal cup positioning [48,49].

Patients in the current study demonstrated a good correction of true LLD, with an average postoperative difference of 0.8 cm between the operated and the contralateral healthy limb. Seven patients required the use of a shoe lift to manage LLD, with 63.1% experiencing a sensation of limb length equality. Conversely, LLD due to adductor contracture required intensive rehabilitation therapy with adductor stretching, and in two patients, an additional percutaneous adductor tenotomy was performed to achieve satisfactory clinical results.

During our retrospective study, we observed three complications: one intraoperative subtrochanteric fracture, one transient paralysis of the femoral nerve, and one superficial surgical wound infection. THA surgery in degenerative arthritis secondary to Crowe IV dislocation is associated with a significant number of complications, including neurovascular compromise due to limb lengthening or direct trauma, intraoperative fractures, component loosening, and dislocation [50,51,52,53]. Patients in the current study showed an overall acceptable complication rate of approximately 16%, and none of these patients required implant revision surgery at the last follow-up. These data are similar or slightly less compared to the complication rate presented in other studies, including those where other approaches and femoral shortening osteotomy were performed, where the risk of non-union is also to be considered [50,51]. Transient paralysis of the femoral or sciatic nerve, although relatively rare, must be carefully considered in patients with severe flexion contracture who undergo intraoperative limb lengthening. In our cohort of patients, one femoral nerve apraxia and no sciatic nerve paralysis were observed. The risk of paralysis increases in case of limb lengthening above 4 cm [54,55] due to traction of the nerve or compression exerted by the post-surgical hematoma. Somatosensory evoked potential (SSEP) was not routinely used during surgery to check for any nerve injuries; however, a careful control of pre- and post-reduction soft tissue tension was performed to avoid nerve traction. In cases of excessive tension or impossibility of reduction, a rescue procedure was performed, such as femoral shortening osteotomy. Moreover, several strategies are employed to minimize this complication, including proximalization of the COR, nerve isolation, and debridement to avoid traction [19,56]. These procedures were not necessary in our patients because, in all cases, it was possible to place the cup at the true acetabulum through soft tissue management. However, it must be emphasized that true LLD greater than 4 cm represented exclusion criteria for the recruitment of patients in the current study. Beyond this elongation limit, our surgical strategy envisages the gradual distalization of the femur using an external fixation device before THA implant.

## 5. Conclusions

In conclusion, THA in the Crowe IV dysplastic hip using DAA allows for excellent clinical and radiographic results with an acceptable complication rate. The approach’s progressive and patient-specific soft tissue release facilitates the positioning of the cup component at the true acetabulum, eliminating the need for femoral shortening osteotomy.

## Figures and Tables

**Table 1:** Pre- and post-operative clinical and radiographic findings.

Variable	Pre-Operative Mean (Range)	Post-Operative Mean (Range)	*p*-Value
Harris Hip Score	44.6 (38–56)	89.4 (82–96)	<0.001
Apparent LLD (cm)	3.5 (2.5–4.3)	1.2 (0.5–2.4)	<0.001
COR (vertical distance, cm)	4.6 (2.8–6.6)	1.9 (1.1–2.4)	<0.002
COR (horizontal distance, cm)	4.4 (4–4.8)	2.4 (1.3–3.4)	<0.0003
True LLD (cm)	3.4 (1.6–4)	0.8 (0.3–1.1)	<0.0001

LLD, limb length discrepancy; COR, center of rotation.

## Data Availability

The data presented in this study are available on request from the corresponding author.

## Supplementary Materials

The following supporting information can be downloaded at: https://www.mdpi.com/article/10.3390/medicina60010114/s1, Video S1: THA by DAA in Crowe IV: surgical technique, Video S2: adductors percutaneous tenotomy.

## References

[1] Husson J.L., Mallet J.F., Huten D., Odri G.A., Morin C., Parent H.F. (2010). Applications in Hip Pathology. Orthop. Traumatol. Surg. Res..

[2] Greber E.M., Pelt C.E., Gililland J.M., Anderson M.B., Erickson J.A., Peters C.L. (2017). Challenges in Total Hip Arthroplasty in the Setting of Developmental Dysplasia of the Hip. J. Arthroplast..

[3] Montalti M., Castagnini F., Giardina F., Tassinari E., Biondi F., Toni A. (2018). Cementless Total Hip Arthroplasty in Crowe III and IV Dysplasia: High Hip Center and Modular Necks. J. Arthroplast..

[4] Li Y., Zhang X., Wang Q., Peng X., Wang Q., Jiang Y., Chen Y. (2017). Equalisation of Leg Lengths in Total Hip Arthroplasty for Patients with Crowe Type-IV Developmental Dysplasia of the Hip. Bone Jt. J..

[5] Sakellariou V.I., Christodoulou M., Sasalos G., Babis G.C. (2014). Reconstruction of the Acetabulum in Developmental Dysplasia of the Hip in Total Hip Replacement. Arch. Bone Jt. Surg..

[6] Bicanic G., Barbaric K., Bohacek I., Aljinovic A., Delimar D. (2014). Current Concept in Dysplastic Hip Arthroplasty: Techniques for Acetabular and Femoral Reconstruction. World J. Orthop..

[7] Pagnano M.W., Hanssen A.D., Lewallen D.G., Shaughnessy W.J. (1996). The Effect of Superior Placement of the Acetabular Component on the Rate of Loosening after Total Hip Arthroplasty: Long-Term Results in Patients Who Have Crowe Type-II Congenital Dysplasia of the Hip. J. Bone Jt. Surg..

[8] Shi X.-t., Li C.-f., Han Y., Song Y., Li S.-x., Liu J.-g. (2019). Total Hip Arthroplasty for Crowe Type IV Hip Dysplasia: Surgical Techniques and Postoperative Complications. Orthop. Surg..

[9] Wu X., Li S.H., Lou L.M., Cai Z.D. (2012). The Techniques of Soft Tissue Release and True Socket Reconstruction in Total Hip Arthroplasty for Patients with Severe Developmental Dysplasia of the Hip. Int. Orthop..

[10] Heuter C. (1883). Grundriss Der Chirurgie. Grundriss der Chirurgie.

[11] Smith-Petersen M.N., Larson C.B. (1947). Complications of Old Fractures of the Neck of the Femur; Results of Treatment of Vitallium-Mold Arthroplasty. J. Bone Jt. Surg. Am..

[12] Putti V., Faldini G., Pasquali E. (1935). Anatomy of Congenital Dislocation of the Hip.

[13] Cadossi M., Sambri A., Tedesco G., Mazzotti A., Terrando S., Faldini C. (2017). Anterior Approach in Total Hip Replacement. Orthopedics.

[14] Lovell T.P. (2008). Single-Incision Direct Anterior Approach for Total Hip Arthroplasty Using a Standard Operating Table. J. Arthroplast..

[15] Mirza A.J., Lombardi A.V., Morris M.J., Berend K.R. (2014). A Mini-Anterior Approach to the Hip for Total Joint Replacement: Optimising Results: Improving Hip Joint Replacement Outcomes. Bone Jt. J..

[16] Parvizi J., Restrepo C., Maltenfort M.G. (2016). Total Hip Arthroplasty Performed Through Direct Anterior Approach Provides Superior Early Outcome: Results of a Randomized, Prospective Study. Orthop. Clin. N. Am..

[17] Nogler M.M., Thaler M.R. (2017). The Direct Anterior Approach for Hip Revision: Accessing the Entire Femoral Diaphysis Without Endangering the Nerve Supply. J. Arthroplast..

[18] Taunton M.J., Trousdale R.T., Sierra R.J., Kaufman K., Pagnano M.W. (2018). John Charnley Award: Randomized Clinical Trial of Direct Anterior and Miniposterior Approach THA: Which Provides Better Functional Recovery?. Clin. Orthop. Relat. Res..

[19] Oinuma K., Tamaki T., Miura Y., Kaneyama R., Shiratsuchi H. (2014). Total Hip Arthroplasty with Subtrochanteric Shortening Osteotomy for Crowe Grade 4 Dysplasia Using the Direct Anterior Approach. J. Arthroplast..

[20] Kawasaki M., Hasegawa Y., Okura T., Ochiai S., Fujibayashi T. (2017). Muscle Damage After Total Hip Arthroplasty Through the Direct Anterior Approach for Developmental Dysplasia of the Hip. J. Arthroplast..

[21] Viamont-Guerra M.R., Chen A.F., Stirling P., Nover L., Guimarães R.P., Laude F. (2020). The Direct Anterior Approach for Total Hip Arthroplasty for Severe Dysplasia (Crowe III and IV) Provides Satisfactory Medium to Long-Term Outcomes. J. Arthroplast..

[22] Viamont-Guerra M.R., Saffarini M., Laude F. (2020). Surgical Technique and Case Series of Total Hip Arthroplasty with the Hueter Anterior Approach for Crowe Type-IV Dysplasia. J. Bone Jt. Surg. Am..

[23] Jawad M.U., Scully S.P. (2011). In Brief: Crowe’s Classification: Arthroplasty in Developmental Dysplasia of the Hip. Clin. Orthop. Relat. Res..

[24] Rodriguez J.A., Kamara E., Cooper H.J. (2017). Applied Anatomy of the Direct Anterior Approach for Femoral Mobilization. JBJS Essent. Surg. Tech..

[25] Banaszkiewicz P.A. (2014). Traumatic Arthritis of the Hip after Dislocation and Acetabular Fractures: Treatment by Mold Arthroplasty: An End-Result Study Using a New Method of Result Evaluation. Classic Papers in Orthopaedics.

[26] Abraham W., Dimon J. (1992). Leg Length Discrepancy in Total Hip Arthroplasty. Orthop. Clin. N. Am..

[27] Sabharwal S., Kumar A. (2008). Methods for Assessing Leg Length Discrepancy. Clin. Orthop. Relat. Res..

[28] Moore M.S., McAuley J.P., Young A.M., Engh C.A. (2006). Radiographic Signs of Osseointegration in Porous-Coated Acetabular Components. Clin. Orthop. Relat. Res..

[29] Engh C.A., Bobyn J.D., Glassman A.H. (1987). Porous-Coated Hip Replacement. The Factors Governing Bone Ingrowth, Stress Shielding, and Clinical Results. J. Bone Jt. Surg. Ser. B.

[30] Komiyama K., Fukushi J.-i., Motomura G., Hamai S., Ikemura S., Fujii M., Nakashima Y. (2019). Does High Hip Centre Affect Dislocation after Total Hip Arthroplasty for Developmental Dysplasia of the Hip?. Int. Orthop..

[31] Della Valle A.G., Padgett D.E., Salvati E.A. (2005). Preoperative Planning for Primary Total Hip Arthroplasty. J. Am. Acad. Orthop. Surg..

[32] Glorion C. (2018). Surgical Reduction of Congenital Hip Dislocation. Orthop. Traumatol. Surg. Res..

[33] Rüdiger H.A., Parvex V., Terrier A. (2016). Impact of the Femoral Head Position on Moment Arms in Total Hip Arthroplasty: A Parametric Finite Element Study. J. Arthroplast..

[34] Hueter C. (1880). Die Methodik Der Resectio Coxae Durch Vorderen Schrägschnitt. (Methode von Schede Und C. Hueter). Verletzungen Und Krankheiten Des Hüftgelenks. Resectio Coxae. Grundriss der Chirurgie.

[35] Rachbauer F., Kain M.S.H., Leunig M. (2009). The History of the Anterior Approach to the Hip. Orthop. Clin. N. Am..

[36] Faldini C., Miscione M.T., Chehrassan M., Acri F., Pungetti C., D’Amato M., Luciani D., Giannini S. (2011). Congenital Hip Dysplasia Treated by Total Hip Arthroplasty Using Cementless Tapered Stem in Patients Younger than 50 Years Old: Results after 12-Years Follow-Up. J. Orthop. Traumatol..

[37] Corten K., Holzapfel B.M. (2021). Direct Anterior Approach for Total Hip Arthroplasty Using the “Bikini Incision”. Oper. Orthop. Traumatol..

[38] Faldini C., Brunello M., Pilla F., Geraci G., Stefanini N., Tassinari L., Di Martino A. (2023). Femoral Head Autograft to Manage Acetabular Bone Loss Defects in THA for Crowe III Hips by DAA: Retrospective Study and Surgical Technique. J. Clin. Med..

[39] Sofu H., Kockara N., Gursu S., Issin A., Oner A., Sahin V. (2015). Transverse Subtrochanteric Shortening Osteotomy During Cementless Total Hip Arthroplasty in Crowe Type-III or IV Developmental Dysplasia. J. Arthroplast..

[40] Mu W., Yang D., Xu B., Mamtimin A., Guo W., Cao L. (2016). Midterm Outcome of Cementless Total Hip Arthroplasty in Crowe IV-Hartofilakidis Type III Developmental Dysplasia of the Hip. J. Arthroplast..

[41] Ahmed E., Ibrahim E.G., Ayman B. (2015). Total Hip Arthroplasty with Subtrochanteric Osteotomy in Neglected Dysplastic Hip. Int. Orthop..

[42] Li X., Lu Y., Sun J., Lin X., Tang T. (2017). Treatment of Crowe Type-IV Hip Dysplasia Using Cementless Total Hip Arthroplasty and Double Chevron Subtrochanteric Shortening Osteotomy: A 5- to 10-Year Follow-Up Study. J. Arthroplast..

[43] Gofton J.P. (1971). Studies in Osteoarthritis of the Hip. IV. Biomechanics and Clinical Considerations. Can. Med. Assoc. J..

[44] Watts C.D., Abdel M.P., Hanssen A.D., Pagnano M.W. (2016). Anatomic Hip Center Decreases Aseptic Loosening Rates after Total Hip Arthroplasty with Cement in Patients with Crowe Type-II Dysplasia: A Concise Follow-up Report at a Mean of Thirty-Six Years. J. Bone Jt. Surg. Am. Vol..

[45] Linde F., Jensen J. (1988). Socket Loosening in Arthroplasty for Congenital Dislocation of the Hip. Acta Orthop. Scand..

[46] Stans A.A., Pagnano M.W., Shaughnessy W.J., Hanssen A.D. (1998). Results of Total Hip Arthroplasty for Crowe Type III Developmental Hip Dysplasia. Clin. Orthop. Relat. Res..

[47] Nawabi D.H., Meftah M., Nam D., Ranawat A.S., Ranawat C.S. (2014). Durable Fixation Achieved with Medialized, High Hip Center Cementless THAs for Crowe II and III Dysplasia. Clin. Orthop. Relat. Res..

[48] Traina F., De Fine M., Biondi F., Tassinari E., Galvani A., Toni A. (2009). The Influence of the Centre of Rotation on Implant Survival Using a Modular Stem Hip Prosthesis. Int. Orthop..

[49] Traina F., De Fine M., Tassinari E., Sudanese A., Calderoni P.P., Toni A. (2011). Modular Neck Prostheses in DDH Patients: 11-Year Results. J. Orthop. Sci..

[50] Wang D., Li L.L., Wang H.Y., Pei F.X., Zhou Z.K. (2017). Long-Term Results of Cementless Total Hip Arthroplasty With Subtrochanteric Shortening Osteotomy in Crowe Type IV Developmental Dysplasia. J. Arthroplast..

[51] Krych A.J., Howard J.L., Trousdale R.T., Cabanela M.E., Berry D.J. (2009). Total Hip Arthroplasty with Shortening Subtrochanteric Osteotomy in Crowe Type-IV Developmental Dysplasia. J. Bone Jt. Surg..

[52] Zhu B., Su C., He Y., Chai X., Li Z., Hou Z., Lou T., Yan X. (2017). Combined Anteversion Technique in Total Hip Arthroplasty for Crowe IV Developmental Dysplasia of the Hip. HIP Int..

[53] Shi X.-t., Li C.-f., Cheng C.-m., Feng C.-y., Li S.-x., Liu J.-g. (2019). Preoperative Planning for Total Hip Arthroplasty for Neglected Developmental Dysplasia of the Hip. Orthop. Surg..

[54] Yang S., Cui Q. (2012). Total Hip Arthroplasty in Developmental Dysplasia of the Hip: Review of Anatomy, Techniques and Outcomes. World J. Orthop..

[55] Edwards B.N., Tullos H.S., Noble P.C. (1987). Contributory Factors and Etiology of Sciatic Nerve Palsy in Total Hip Arthroplasty. Clin. Orthop. Relat. Res..

[56] Kawai T., Tanaka C., Kanoe H. (2014). Total Hip Arthroplasty for Crowe IV Hip without Subtrochanteric Shortening Osteotomy—A Long Term Follow up Study. BMC Musculoskelet. Disord..

